# Risk Assessment and Hazardous Effects of Metal Contamination in *O. niloticus* and *S. galilaeus* from Four Islands of the River Nile

**DOI:** 10.1007/s00128-022-03589-1

**Published:** 2022-08-07

**Authors:** Engy Elhaddad, Sally M. Salaah, Hanan M. M. Salama, Dina M. El-Sherif, Hanan S. Gaber

**Affiliations:** National Institute of Oceanography and Fisheries (NIOF), Cairo, Egypt

**Keywords:** The Nile River islands, The Great Cairo sector, Pollution, Health risk, Histopathological alterations, Metal load

## Abstract

The Nile River islands are recognized as the most productive lands in Egypt. Although, these islands are vulnerable to several natural and man-made crises. The present study was aimed to evaluate the consequences of different anthropogenic activities on the heavy metals load and histological alterations in *O. niloticus* and *S. galilaeus* collected from four different Nile River islands along the Great Cairo sector (Egypt), and the possible health risks for human consumers. Metals were accumulated in both fish muscles in the following order: Fe > Zn > Cu > Mn > Pb. *S. galilaeus* was recorded higher metal pollution index than *O. niloticus*, while El-Warrak Island was documented the highest MPI and hazard quotient among all sampling sites. All sampled tissues were recorded histopathological lesions in both fish. The present study may be considered as an early alert for habitual consumers, particularly at high consumption rates of some fish species.

The Nile River in Egypt is the main freshwater resource, with more than ninety percent of the population living nearby or directly relying on it. Thus, it is continuously exposed to excessive anthropogenic activity such as; municipal, industrial, wastewater, and agricultural which have stimulated a dramatic decline in water quality (Abd Ellah 2020). For instance, the Nile River from Aswan to El-Kanater Barrage receives wastewater discharge from about 67 agricultural drains and 57 industrial sources (Elewa 2010; Elhaddad and Al-Zyoud 2017), of which ten drains only meet the Egyptian standards of the drainage water discharged into the Nile (Ezzat et al. 2002).

The Great Cairo is the main urban area of Egypt and Africa (CAPMAS 2021). This growing population was followed by an increase in urbanization, modern industries, agricultural, and touristic activities. The Nile River receives a massive quantity of polluted water while crossing the Great Cairo, which intimidates the health of both humans and aquatic animals (Salaah and El-Gaar 2020).

There are about 144 islands scattered along the main course of the Nile River from Aswan to the Delta region. The Nile River islands are recognized as the most productive lands in Egypt, some rest stops for migratory birds, while others have unique features of land surfaces with vegetation and animal diversity, which is considered to be rare in other environments. The clean dry climate of these islands made them a favorable destination for tourists from all over the world (Farag and El-Alfy 2013).

The Nile River islands may be categorized into four main practices; residential islands, agricultural islands, tourist islands, and deserted islands (Zalalu 1989). However, due to their fertile soils, many of the Nile River islands have been inhabited and cultivated, as a result, they are highly vulnerable to the negative impact of anthropogenic actions (Barron 2006).

Although, The Nile River islands have significant characteristics as compared to other river islands around the world. Recently, the Nile islands suffer from many issues such as; land cultivation, uncontrolled urbanization, and tourism activities, overpopulation, lack of awareness programmers. Besides, the agricultural land erosion owing to urban expansion and meager farming practices (Amer 2009). It is worth mentioning that, the Nile River islands did not receive appropriate research to identify the impact of water pollutants such as heavy metals associated with the anthropogenic activity on aquatic animals and human risk.

The Aquatic ecosystems face many threats include pollution, among different types of water contaminants, heavy metals (HM) are a particularly serious ecological problem for humans and ecosystems owing to their abundance, toxicity, insistence, and accumulation capacity (Goher et al. 2014).

In Egypt, fish is considered the first and cheapest source of protein, however fish can accumulate HM in their organs in concentrations that exceed many times the available in their surroundings (Abdel-Khalek et al. 2016; Salaah and El-Gaar 2020). In fish, HM can persuade many biological and histological irreversible responses which their health status (Gaber 2008; Khalil et al. 2017; Salaah et al. 2018).

Histopathological alterations are used as a gadget in the ecological hazard estimation and monitoring plans induced by anthropogenic pollutants, which reflect the overall health of the whole population within the ecosystem (El-Naggar et al. 2019).

Nowadays, Egypt faces a scarce of water besides the change in water quality due to the Grand Ethiopian Renaissance Dam filling. The present work is the first conducted study after the second phase of the Grand Ethiopian Renaissance Dam filling. The present work aimed to (1) Deliver comparable data of heavy metals accumulation associated with histopathological changes in tissues of *O. niloticus* and *S. galilaeus* collected from inhabited four islands along the Great Cairo sector during spawning season. (2) Assess the possible human health risk related to fish consumption. The chosen locations are appropriate for assessing the impacts of anthropogenic activities on metal contamination in the Nile River main stream.

## Materials and Methods

Four islands of the Nile River located at the great Cairo sector in Egypt were chosen to characterize the impact of heavy metals induced by different types of anthropogenic activity during summer 2020 (Table 1). S1 (El-Manial Island) is the smallest island with an area of about 2.1 km^2^ with 3.2 km length and 0.7 km width. S2 (El-Zamalek Island) with an area of 3.6 km^2^ with 3.9 km length and 0.9 km width. S3 (El-Warrak Island) is the biggest island with an area of 7.3 Km^2^ with 5 km length and 1.7 km width. S4 (Alkiratian Island) is 3.8 km length and 0.9 km width with an area of 2.6 Km^2^. Where S1 and S2 demonstrated the residential and touristic activities impact, S3 represented both agricultural and residential activities, While S4 delivered the impact of agricultural activity.

**Table 1:** Latitude and longitude of the study sites from the Nile River islands along the Great Cairo sector

Location	S1 (El-Manial)		S2(El-Zamalek)		S3 (El-Warrak)		S4 (El-Kerateen)	
	Latitude	Longitude	Latitude	Longitude	Latitude	Longitude	Latitude	Longitude
1	30° 00′ 23.1′′	31° 13′ 29.7′′	30° 02′ 14.5′′	31° 13′ 25.2′′	30° 05′ 33.5′′	31° 13′ 53.1′′	30° 09′ 05.3′′	31° 09′ 23.0′′
2	30° 00′ 55.1′′	31° 14′ 40.8′′	30° 02′ 59.5′′	31° 13′ 42.9′′	30° 06′ 35.4′′	31° 14′ 22.4′′	30° 09′ 34.2′′	31° 09′ 04.1′′
3	30° 01′ 43.9′′	31° 13′ 49.9′′	30° 03′ 37.2′′	31° 13′ 32.0′′	30° 07′ 48.5′′	31° 13′ 05.7′′	30° 10′ 04.7′′	31° 08′ 37.1′′
4	30° 00′ 59.7′′	31° 13′ 16.3′′	30° 03′ 00.9′′	31° 13′ 05.4′′	30° 06′ 24.6′′	31° 13′ 15.8′′	30° 09′ 21.0′′	31° 08′ 40.0′′
5	30° 01′ 43.8′′	31° 13′ 21.3′′	30° 03′ 48.8′′	31° 12′ 54.6′′	30° 07′ 13.0′′	31° 12′ 49.5′′	30° 09′ 54.6′′	31° 07′ 57.0′′
6	30° 02′ 09.7′′	31° 13′ 40.4′′	30° 04′ 25.5′′	31° 13′ 22.5′′	30° 07′ 55.2′′	31° 11′ 48.6′′	30° 10′ 33.0′′	31° 07′ 36.3′′

The present study was conducted using the stratified random sampling method, since it is typically designed to be size-selective and species-specific. Fish samples were collected from six locations around each island (three at each bank-side-of the lake). A total of 288 fish [144 samples of each Nile tilapia (*O. niloticus*) and Mango tilapia (*S. galilaeus*) were assembled with the help of resident fishermen]. Six fish of each fish species were collected from each sampling site. Fish length and weight were 27.5 ± 2.6 cm and 165 ± 24.7 g for both *O. niloticus* and *S. galilaeus*. Fish were dissected to obtain muscles samples for heavy metals analysis (n = 36). Tissues were dried and acid digested, then filtered and transferred to a plastic tube, deionized water was added to the appropriate volume according to Ghazaly (1988). Heavy metals in fish muscles were analyzed using atomic absorption (GBC Savanta AA AA with GF 5000 graphite furnace).

Analytical blanks, triplicate analyses, standard solutions made in the same acid matrix, and standard reference material were all used in the QA/QC processes. Instrument calibration standards were created using a certified mono-element efference solution (Merck). The metal average recovery percentages for all analyzed samples ranged from 93% to 107% using standard reference material (Lake Superior fish 1946; National Institute of Standards and Technology (NIST), USA).

MPI was used to study the capacity of fish to accumulate HM using the calculation of Usero et al. (1997):

$${\mathrm{MPI}= \left(\mathrm{Cf}1 \times \mathrm{ Cf}2 \times \mathrm{ Cf}3 \times \dots \mathrm{ Cfn}\right) }^{1/n}$$

where, Cf*n* is the concentrations for the metal *n* (mg/kg dry weight) in the sample.

According to USEAP (1989), the ingestion dose of HM polluted food equals the absorbed dose. So, the estimated daily intake of HM (EDI), target hazard quotients (THQ), hazard index (HI), and target cancer risk (TR) were considered.

EDI is measured according to Song et al. (2009) and expressed as mg/kg body-weight/day.

$$\mathrm{EDI}=\frac{\left(\mathrm{Mc }\times \mathrm{ IR}\right)}{\mathrm{BW}}$$

where Mc is the concentration of a single metal in fish tissue, IR is the ingestion rate (kg per day), BW is the body weight of normal adults.

The THQ is used to evaluate the level of non-carcinogenic risk as a result of exposure to particular toxins according to USEPA (2011);

$$\mathrm{THQ}=\frac{\left(\mathrm{Mc }\times \mathrm{ IR }\times \mathrm{ EF }\times \mathrm{ ED}\right)}{(\mathrm{RfD} \times \mathrm{ BW }\times \mathrm{ ATn})}$$

where EF is the exposure frequency, ED is the exposure duration, RfD is the reference dose of according to USEPA (2012), ATn is the meantime of exposure to non-carcinogens.

HI used to determine the possible health risk posed by metals according to USEPA (2011).

TR is used to predict the carcinogenic risk (USEAP 2011).

$$\mathrm{TR}=\frac{\left(\mathrm{Mc }\times \mathrm{ IR }\times \mathrm{ CPSo }\times \mathrm{ EF }\times \mathrm{ ED}\right)}{(\mathrm{BW }\times \mathrm{ ATc})},$$

where CPSo is the carcinogenic potency slope of oral Pb according to USEPA (2012). ATc is the averaging time for carcinogens.

Specimens from different organs (spleen, liver, and gonad (ovary and testis) of *O. niloticus* and *S. galilaeus* were collected for the histological examinations from each site of the Nile River along the Great Cairo sector. Specimens from different organs were fixed in neutral formalin (10%), dehydrated, embedded in paraffin wax, and sectioned at 4–6 µm thin, stained with hematoxylin and eosin, and examined microscopically (Bernet et al. 1999).

Data of heavy metal assessment were collected as a replica (N = 36) for each sampling island. One-way analysis of variance (ANOVA) was used to analyze the data using the statistical package for social sciences (SPSS 2013). Data were considered significant at *p* < 0.05, then Tukey’s post hoc tests were used to identify variances among different sites.

## Results

Table 2 represents the concentrations pattern of different heavy metals (HM) in both *O. niloticus* and *S. galilaeus* muscle were: Fe > Zn > Cu > Mn > Pb in both fish, while *S. galilaeus* showed higher a tendency to accumulate HM than *O. niloticus,* among all sampling sites. Fe concentrations in both fish recorded significant (*p* ≤ 0.05) changes among sites. In *S. galilaeus*, Fe accumulation was increased by 1.7, 1.1, 1.2, and 1.2 folds than *O. niloticus* at S1, S2, S3, and S4, respectively. Mn concentrations at S3 were significantly increased (*p* < 0.05) in both fish as compared to other sites during the study. Levels of Mn in *S. galilaeus were* increased by 1.4, 1.5, 1.2, and 1.3 than *O. niloticus* at sampling sites in order.

**Table 2:** Heavy metals concentration (mg/kg drywt) and metals pollution index (MPI) of *O. niloticus* and *S. galilaeus* muscles from different Nile River islands along the Great Cairo sector

	S1	S2	S3	S4	Permissible limits
Fe					
*O. niloticus*	12.3 ± 0.33^c^	6.98 ± 0.44^d^	70.63 ± 4.19^a^	47.24 ± 2.32^b^	100^AB^
*S. galilaeus*	21.67 ± 1.57^c^	8.0 ± 0.31^d^	81.56 ± 2.82^a^	58.17 ± 1.49^b^	
Mn					
*O. niloticus*	0.46 ± 0.04^c^	0.20 ± 0.49^c^	1.89 ± 0.14^a^	1.06 ± 0.77^bc^	1^A^
*S. galilaeus*	0.63 ± 0.08^c^	0.29 ± 0.03^d^	2.28 ± 0.38^a^	1.33 ± 0.01^b^	
Zn					
*O. niloticus*	21.19 ± 1.31^c^	4.93 ± 0.36^d^	47.17 ± 2.27^a^	25.33 ± 1.11^b^	100^A^
*S. galilaeus*	27.14 ± 3.26^c^	6.66 ± 0.95^d^	53.71 ± 2.89^a^	31.95 ± 0.70^b^	75^B^
Cu					
*O. niloticus*	2.75 ± 0.33^c^	1.62 ± 0.37^d^	7.71 ± 0.24^a^	4.47 ± 0.43^b^	30^A^
*S. galilaeus*	3.16 ± 0.25^c^	2.55 ± 0.60^d^	8.34 ± 0.15^a^	6.05 ± 0.22^b^	
Pb					
*O. niloticus*	0.08 ± 0.00^c^	0.02 ± 0.00^c^	1.11 ± 0.13^a^	0.26 ± 0.02^b^	0.5^A^
*S. galilaeus*	0.10 ± 0.01^d^	0.03 ± 0.00^c^	1.37 ± 0.07^a^	0.39 ± 0.04^b^	0.2^B^
MPI					
*O. niloticus*	1.95	0.77	8.84	4.32	
*S. galilaeus*	2.61	1.07	10.27	5.60	

All values are given as mean ± SD (n = 36). Values within a row with different superscripts differ significantly (Tukey’s Multiple Range Test, *p* < 0.05)

^A^FAO/WHO (1989)

^B^FEPA (2003)

Zn concentration in both fish showed a significantly different (*p* ≤ 0.05) among sampling sites, while S3 recorded the highest Zn levels. Zn was accumulated in muscles of *S. galilaeus* more than *O. niloticus* by 1.3, 1.4, 1.1, and 1.3 folds at sampling sites in order. Table 2 displayed a remarkable increase (*p* ≤ 0.05) at S3 in Cu concentration in *O. niloticus* and *S. galilaeus* muscles among sampling sites during the present study, Cu levels in *S. galilaeus* exhibited higher levels than *O. niloticus* by 1.1, 1.6, 1.0, and 1.4 according to sites order.

Moreover, S3 recorded a significant increase (*p* ≤ 0.05) in Pb concentrations during the present study, as compared to other sites. *S. galilaeus* accumulated higher Pb than *O. niloticus* by 1.3, 1.5, 1.2, and 1.5 at sites in order, respectively. According to Table 2, the accumulation of Pb was above the recommended guidelines of FAO/WHO (1989) at S3 and the limits of FEPA (2003) at S3 and S4 in muscles of both fish, so the human risk assessment was considered to estimate the potential risk of these toxicants.

The MPI of HM in *O. niloticus* and *S. galilaeus* muscles are displayed in Table 2. MPI of HM were in the following pattern: S3 > S4 > S1 > S2. The MPI in the present study was site and species dependent, where MPI in *S. galilaeus* was higher than *O. niloticus* by 1.3, 1.4, 1.2, and 1.3 folds at sites in order.

The estimated daily intake (EDI) of HM from consuming both fish by a normal and habitual adult were represented in Table 3. The EDI of both fish was within the permissible provisional tolerable daily intake (PTDI) values according to USEPA (2014) and Hang et al. (2009). *S. galilaeus* recorded higher EDI among metals than *O. niloticus*, especially for habitual consumers. The target hazard quotient (THQ) in the present study was recorded at S4 (0.932) for Pb habitual consumers of *S. galilaeus* fish (Table 4). In the present study, all considered HM were less than 1, however, in both fish, Pb recorded the highest THQ pursued by Cu > Zn > Fe > Mn, though S4 was the highest THQ followed by S4 > S1 > S2 (Table 4).

**Table 3:** Estimated daily intake (EDI, mg/Kg BW/day) of heavy metals from *O. niloticus* and *S. galilaeus* normal and habitual consumption

	S1	S2	S3	S4
Fe				
*O. niloticus*				
Normal	0.005	0.003	0.031	0.021
Habitual	0.026	0.014	0.143	0.096
*S. galilaeus*				
Normal	0.009	0.003	0.036	0.02
Habitual	0.044	0.016	0.165	0.118
Mn				
*O. niloticus*				
Normal	2.0E−04	9.1E−05	8.4E−04	4.7E−04
Habitual	9.3E−04	4.1E−04	0.003	0.002
*S. galilaeus*				
Normal	2.8E−04	1.2E−04	0.001	5.9E−04
Habitual	0.001	5.7E−04	0.004	0.002
Zn				
*O. niloticus*				
Normal	0.009	0.002	0.021	0.011
Habitual	0.043	0.010	0.095	0.051
*S. galilaeus*				
Normal	0.012	0.002	0.023	0.014
Habitual	0.055	0.013	0.109	0.064
Cu				
*O. niloticus*				
Normal	0.001	7.2E−04	0.003	0.001
Habitual	0.005	0.003	0.015	0.009
*S. galilaeus*				
Normal	0.001	0.001	0.003	0.002
Habitual	0.006	0.005	0.016	0.012
Pb				
*O. niloticus*				
Normal	3.5E−05	1.0E−05	4.9E−04	1.1E−04
Habitual	1.6E−04	4.8E−05	0.002	5.3E−04
*S. galilaeus*				
Normal	4.6E−05	1.6E−05	6.1E−04	1.7E−04
Habitual	2.1E−04	7.4E−05	0.002	7.9E−04

**Table 4:** Hazard quotient (THQ) values of heavy metals from *O. niloticus* and *S. galilaeus* normal and habitual consumption

	S1	S2	S3	S4
Fe				
*O. niloticus*				
Normal	0.008	0.004	0.045	0.030
Habitual	0.038	0.020	0.205	0.137
*S. galilaeus*				
Normal	0.014	0.005	0.052	0.037
Habitual	0.063	0.023	0.237	0.169
Mn				
*O. niloticus*				
Normal	0.001	0.001	0.005	0.003
Habitual	0.006	0.002	0.023	0.013
*S. galilaeus*				
Normal	0.002	0.001	0.006	0.003
Habitual	0.008	0.003	0.027	0.016
Zn				
*O. niloticus*				
Normal	0.031	0.007	0.070	0.038
Habitual	0.144	0.033	0.320	0.172
*S. galilaeus*				
Normal	0.040	0.010	0.080	0.047
Habitual	0.184	0.045	0.364	0.217
Cu				
*O. niloticus*				
Normal	0.031	0.018	0.086	0.050
Habitual	0.140	0.083	0.392	0.228
*S. galilaeus*				
Normal	0.035	0.028	0.093	0.067
Habitual	0.161	0.130	0.424	0.308
Pb				
*O. niloticus*				
Normal	0.012	0.004	0.166	0.039
Habitual	0.054	0.016	0.756	0.180
*S. galilaeus*				
Normal	0.016	0.005	0.204	0.058
Habitual	0.071	0.025	0.932	0.264

Like THQ, HI shouldn’t exceed 1, otherwise, adverse health effects will be expected. At S3, habitual consumer of both fish recorded HI > 1, this indicates possible adverse health effects (Table 5). The target cancer risks (TR) for Pb were calculated according to the available carcinogenic potency slope factors (CSFo) to assess the risk of consuming fish exposed to HM (Baki et al. 2018). The results of the present study detected a negligible risk of Pb (TR ≤ 10^–6^) in *O. niloticus* and *S. galilaeus* for both types of consumers (Table 6).

**Table 5:** Hazard index (HI) posed by heavy metals of *O. niloticus* and *S. galilaeus* normal and habitual consumption

	S1	S2	S3	S4
*O. niloticus*				
Normal	0.08	0.03	0.37	0.16
Habitual	0.38	0.16	1.70*	0.73
*S. galilaeus*				
Normal	0.11	0.05	0.43	0.21
Habitual	0.49	0.23	1.99*	0.97

*Adverse health effects are expected (HI ≥ 1.0)

**Table 6:** Target cancer risk (TR) of lead (Pb) in *O. niloticus* and *S. galilaeus* for normal and habitual consumption

	S1	S2	S3	S4
*O. niloticus*				
Normal	3.0E−07	9.1E−08	4.2E−06	1.0E−06
Habitual	1.3E−06	4.1E−07	1.9E−05	4.6E−06
*S. galilaeus*				
Normal	3.9E−07	1.4E−07	5.2E−06	1.5E−06
Habitual	1.8E−06	6.3E−07	2.4E−05	6.7E−06

The oral carcinogenic slope factor (CSFo) for Cd and Pb is; 0.38 and 0.0085

TR is defined as; low (TR ≤ 10^–6^); moderate (TR from 10^−4^ to 10^−3^); high; (TR from 10^−3^ to 10^−1^); very high (TR ≥ 10^−1^)

**Fig. 1:**
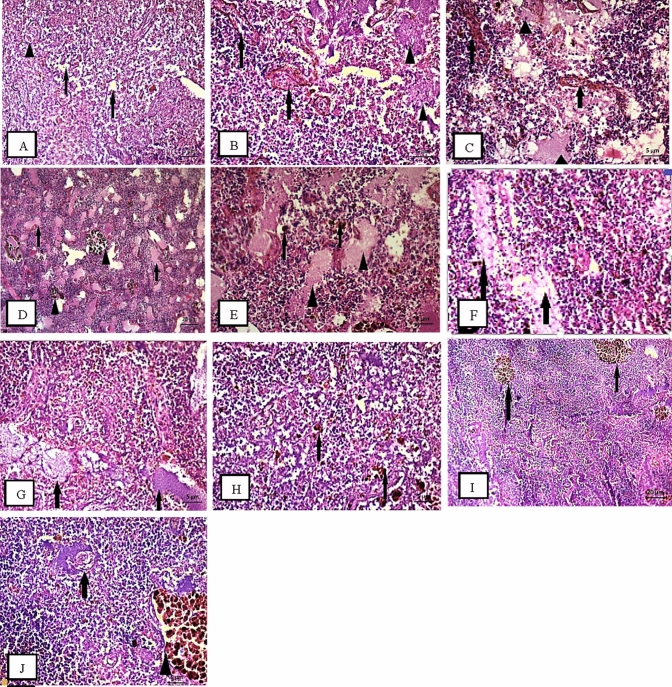
**A** Spleen of *O. niloticus* fish from S1 reported necrotic change in hematopoietic tissue (arrows) and parasite (triangle); **B** severe degeneration (arrows) hemolysis in hematopoietic tissue (triangle); **C** severe hemolysis (arrows) and necrotic change (triangle); **D** spread MMC bundles (triangle) and severe focal areas of hyaline degeneration (arrows); **E** hemosiderin (arrows) and hyaline degeneration (triangle). S2 **F** showed mild spread of melanomacrophage (MMc). S3 **G** recorded hyaline degeneration (arrows) and necrotic change of hematopoietic tissue (arrows). S4 **H** spleen showed spread bundles of MMC; **I** parasite (arrow); **J** parasite (arrow) and bundle of MMC (triangle)

**Fig. 2:**
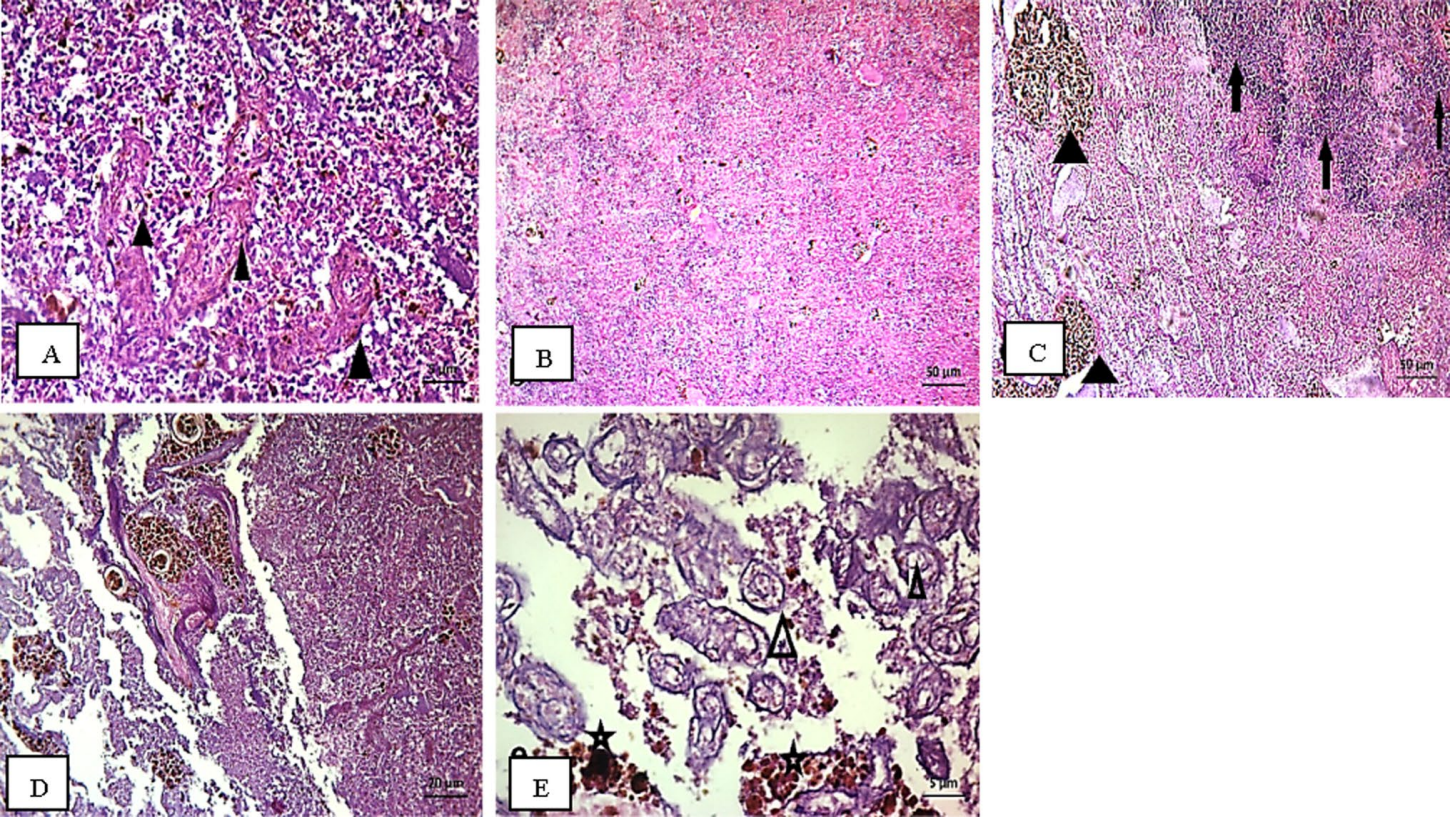
Spleen of fish *S. galilaeus* at S1 showing connective tissue fibers, disorganization of the splenic cords (**A**); S2 recorded red and white pulp appeared nearly like control (**B**); S3 documented MMC (triangle) and tumor (arrows) (**C**); S4 showed MMC aggregates in bundles and parasites (**D** and **E**)

The histopathological analysis revealed spleen lesions in both fish (*O. niloticus* and *S. galilaeus*), the most displayed alterations were recorded in fish from S3 (Figs. 1 and 2), followed by S4 and S1, while S2 was close to control spleen tissue. In Fig. 2, the disorganization of the splenic cords resulted in lymphatic tissue displacement within the substance of splenic pulp and hemosiderin (Fig. 2). Also, loosely packed red and white pulp (necrotic change) were displayed with collapsed melanomacrophage centers (MMCs), which suggested a bacterial infection. Many MMCs groups were observed in both fish (Figs. 1 and 2), besides parasites were detected in *S. galilaeus* (Fig. 2).

**Fig. 3:**
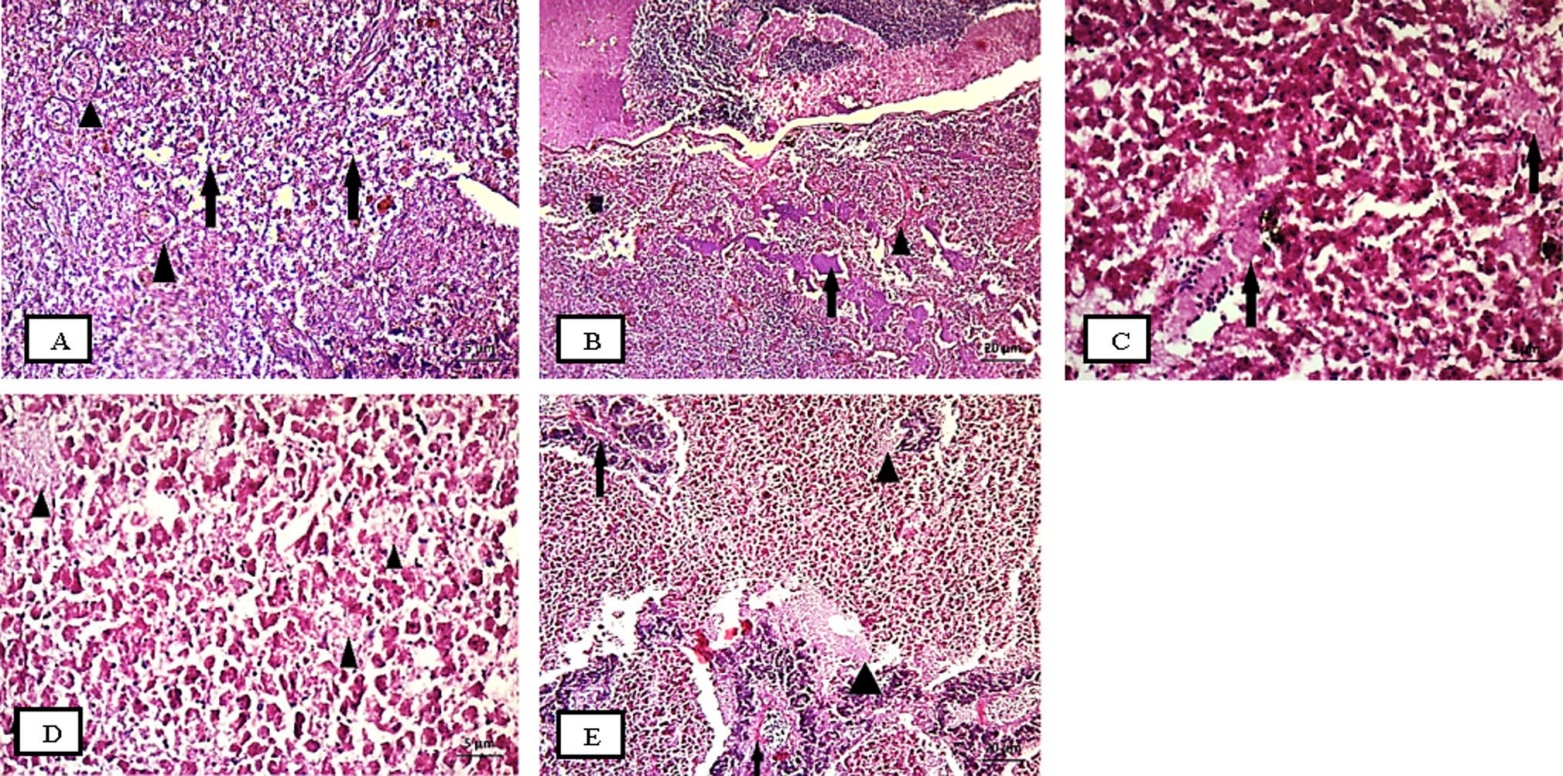
Liver of fish *O. niloticus* from S1 showed hemorrhage between the hepatocytes and parasite (triangle) and necrotic change (arrows) (**A**); hemorrhage (triangle) and hyaline degenerations between the hepatocytes (arrow) (**B**); hemorrhage and hyaline degenerations between the hepatocytes with inflammatory cells infiltration (arrow) (**C**); S3 recorded coagulative necrosis and focal areas of necrosis triangle (**D**); S4 demonstrated dilation and congestion in hepatoportal blood vessel (arrows) and focal areas of necrosis (triangle) (**E**)

**Fig. 4:**
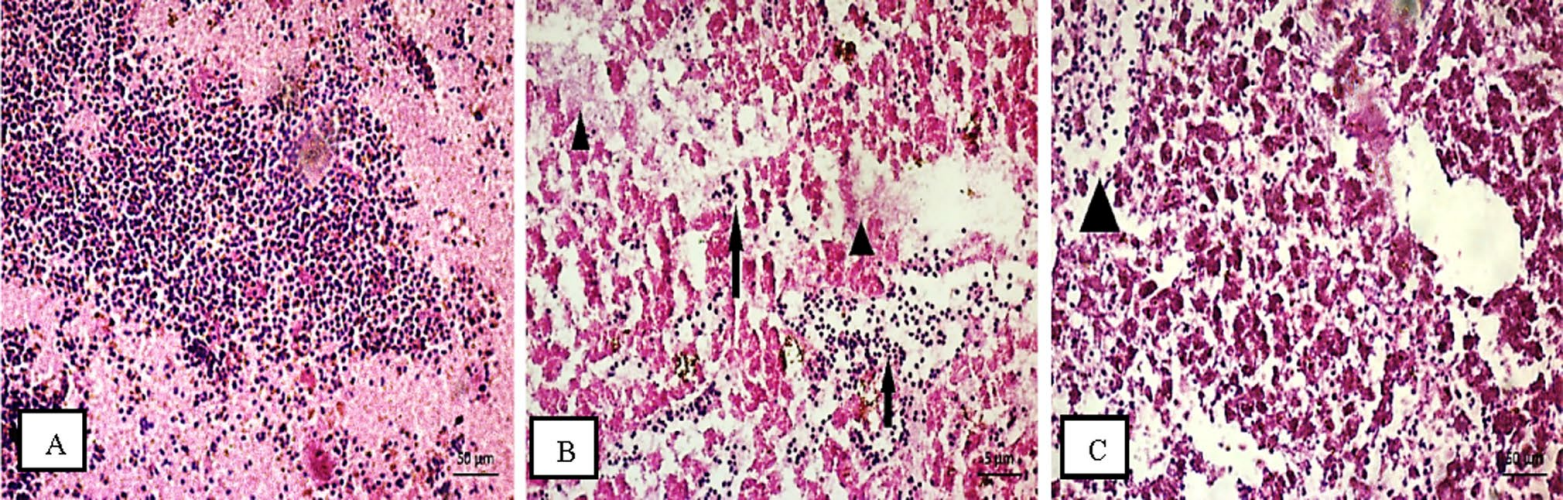
Liver of fish *S. galilaeus* showing, fish from S1 demonstrated severe autolysis with aggregations of inflammatory cells (**A**); S3 with hemorrhage, hemolysis, aggregations of inflammatory cells (arrows) necrotic change (triangle) (**B**); S4 recorded coagulative necrosis and intravascular hemolysis in hepatoportal blood vessel monocytic leukemia (**C**)

The liver of both fish showed hemorrhage and congestion in the hepatic sinusoids along with hepatic vessels dilation. Vacuolar degeneration and fatty changes were observed in hepatocytes (Figs. 3 and 4). In *O. niloticus*, most pancreatic acini were atrophied and showed degenerative changes (Fig. 3). *S. galilaeus* showed severe liver alterations, severe necrotic change, intravascular hemolysis, and thrombosis formation with a stasis of blood displayed pyknotic and karyolytic nuclei, besides inflammatory cells aggregation than in *O. niloticus* (Fig. 4).

**Fig. 5:**
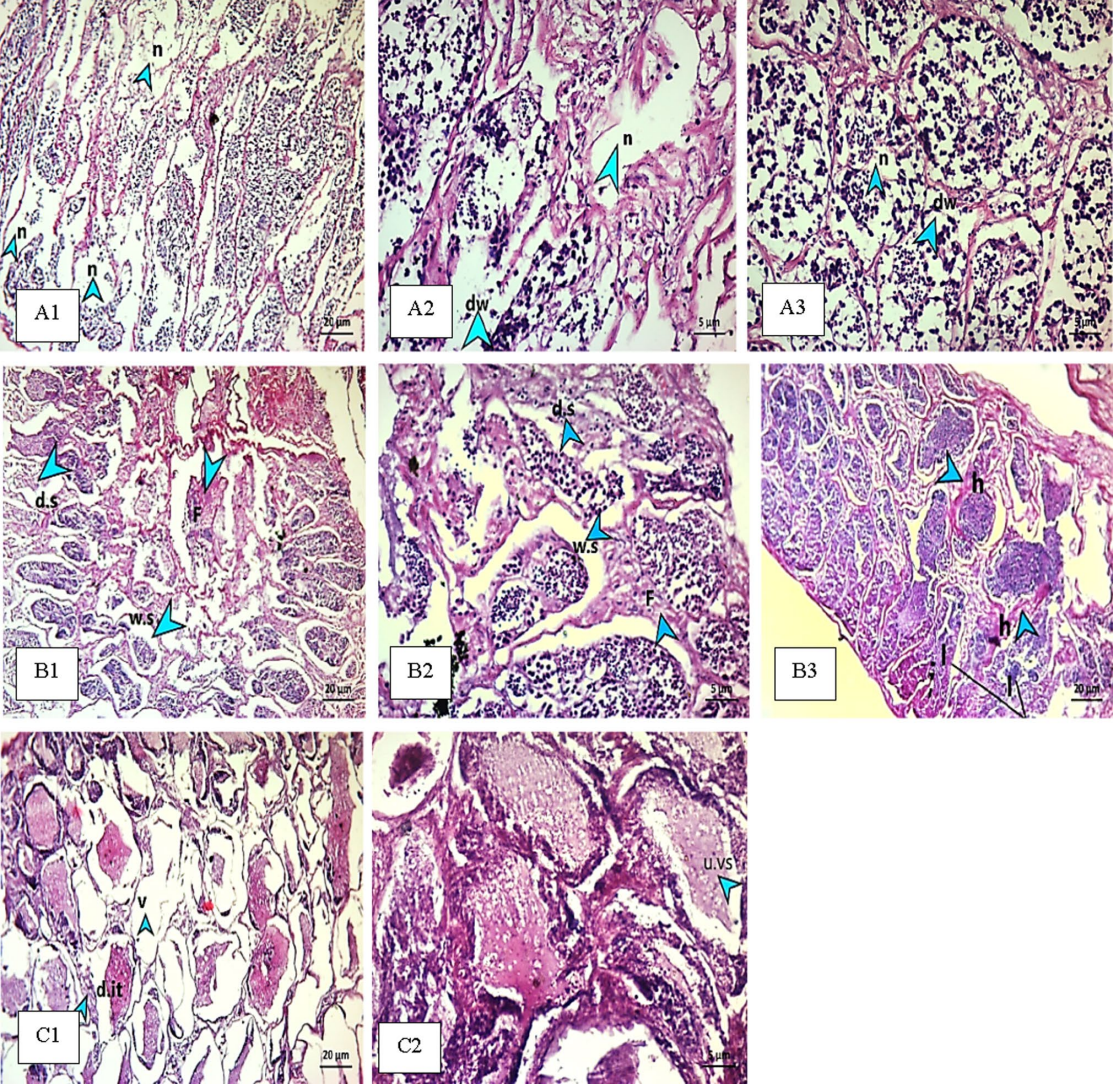
Testes of *O. niloticus* and *S. galilaeus* at S1 showed necrosis (n) (head of arrows) and degeneration of wall (dw) of seminiferous lobules (**A1**, **A2**, **A3**); S3 recorded degeneration of Spermatozoa(ds) (head of arrows), degeneration in surrounding wall of seminiferous lobules (dw) and fibrosis (**B1**, **B2**), hemorrhage (h) and lysis(l) (head of arrows) (**B3**); S4 demonstrated (Vacuolization) (v) (head of arrows) unvital sperms (u.vs) and degeneration of interstitial tissues (d.it) (**C1**, **C2**)

Testes of both fish showed nearly similar histological alternations (Fig. 5). Among all sampling sites, S4 recorded the most pronounced effect in testes of fish followed by S1 and S3. At S4, spermatocytes showed many empty and abnormal shape seminiferous lobules, magnified figure illustrated dead spermatozoa, degeneration of interstitial tissues which surround seminiferous lobules (Fig. 5C1 and C2). At S3, an extensive testicular degeneration was recorded, which caused localized or generalized loss of the germinal epithelium seminiferous lobules, vacuoles in most of germ cells stages, interstitial cells aggregate occupy and expand some interstitial spaces along with hemorrhage (Fig. 5B1, B2, and B3).

**Fig. 6:**
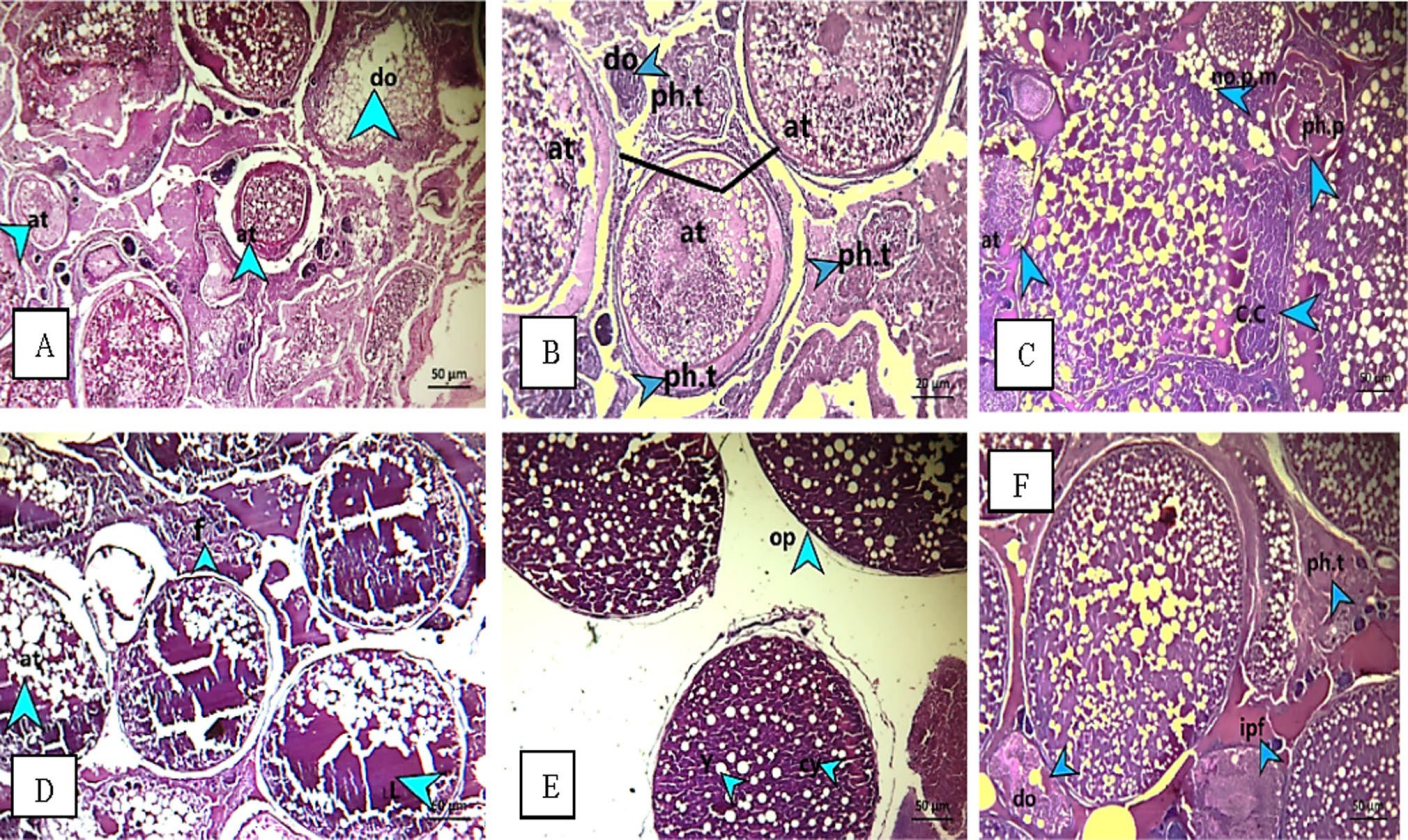
Sections of ovaries of *O. niloticus* at S1 showing degeneration of oocytes (do), atresia. The presence of yolk material (they are late phase of atretic follicles) (at) and phagocytic tissues (internal and external ph.t) (head of arrow) (**A**, **B**), besides carcinogenic case (c.c), cell lost plasma membrane (no p.m), degeneration oocyte (do) and atresia (at) (head of arrow) (**C**); S2 recorded yolk lysis (l) and fibrosis tissues (f) and atresia (at) (head of arrow) (**D**), and very thin ooplasm, yolk droplet and abnormal cytoplasm (head of arrow) (**E**); S4 demonstrated interstitial proteinaceous fluid (ipf), phagocytic tissues (ph.t) and degeneration oocyte (do) (head of arrow) (**F**)

**Fig. 7:**
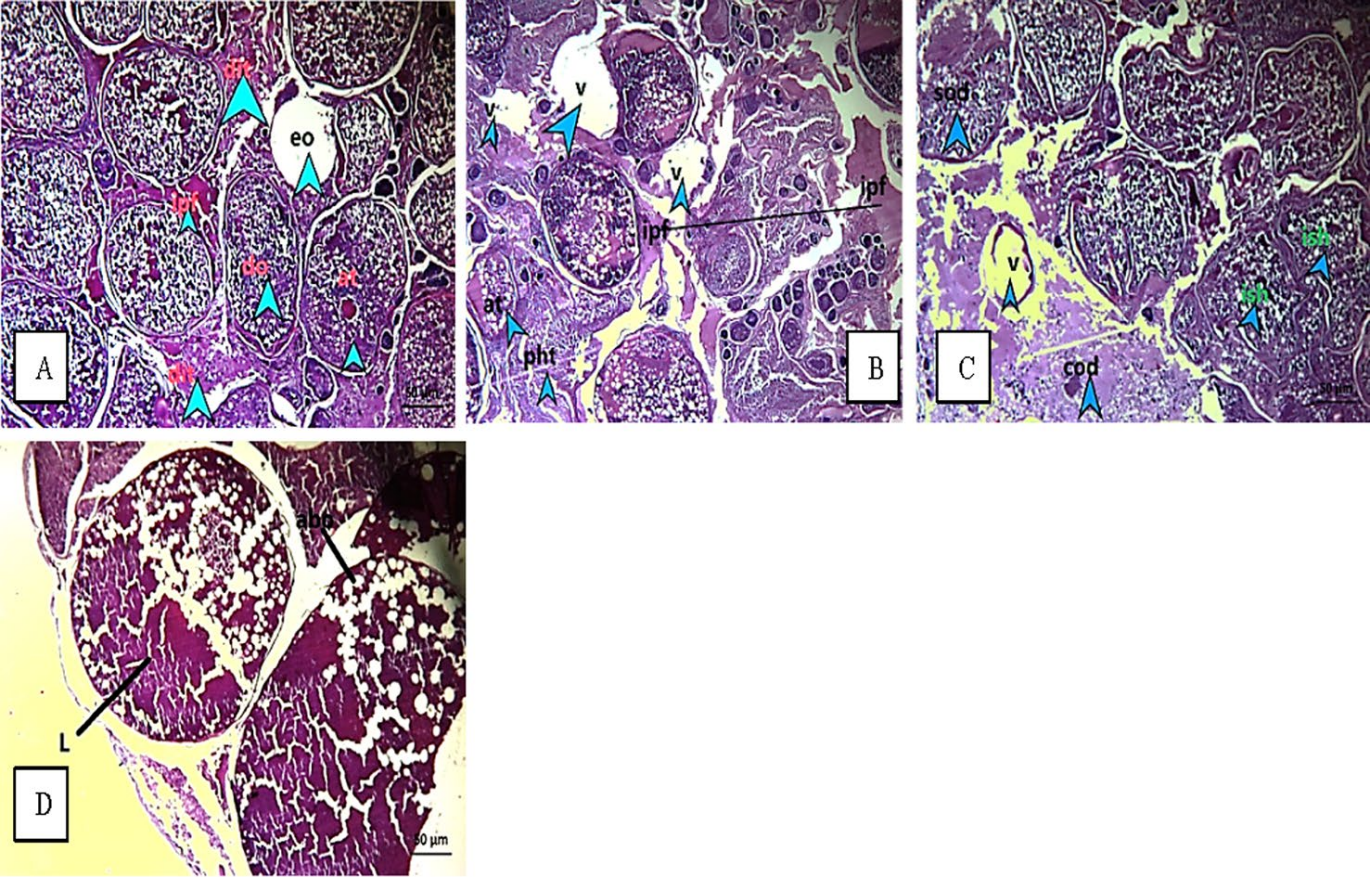
Sections of ovaries of *S. galilaea* at S1 showing interstitial proteinaceous fluid (ipf), empty oocyte (eo) and vacuoles (v), degenerated oocytes (do), completely degeneration to some oocytes (cdo), semi degeneration (sdo), irregular shape of oocytes(i.sh), a collapsed follicle (head of arrow) (**A**, **B**, **C**); S3 demonstrated abnormal oocyte (abo) and lysis(l) (black rode) (**D**)

The sampled ovarian tissues were in previtellogenic and vitellogenic stages during spawning season for both fish. Ovary of *O. niloticus* showed Phagocytic tissues (interstitial tissues) which occupied the section and invade the oocytes. At S1, a degeneration and atresia were recorded in all oocyte’s stages (Fig. 6A–C). the fibrous connective tissues were increased as well as interstitial proteinaceous fluid along with oocytes degeneration in fish ovaries at S2 and S4 (Fig. 6D and E), S2 also illustrated an individual mature oocyte with very thin ooplasm and absorbed yolk droplets (Fig. 6F). On the other hand, S1 and S3 showed the most eminent effects in the ovaries of *S. galilaeus*. Significant degeneration and histological alterations features were shown, as well as degradation and resorption in oocytes at developmental stages (Fig. 7). Ovaries of *S. galilaeus* from S1 displayed much vascular or interstitial proteinaceous fluid and homogenous dark pink translucent material, whereas the interstitial areas may be thickened by the fluid (Fig. 7A–C), while at S3 oocytes were semi and completely degenerated with an irregular shape and lysis of cytoplasm (Fig. 7D).

## Discussion

Recently, water pollutants have become a serious hazard, especially in the growing population. Urbanization and industrialization have increased the discarding of pollutants like HM into the environment. Metals are the most hazardous pollutant for aquatic animals, due to their tolerance even at low concentrations, and being extremely virulent for humans.

The present study demonstrated the impact of anthropogenic activities, which induce an abundance of metals in aquatic systems. Fish are so sensitive to environmental fluctuations; thus, fish has been used as an indicator of metal pollution in aquatic systems (Khalil et al. 2017). Fish from heavily polluted areas will be more affected than others (Omar et al. 2015). Avigliano et al (2019) reported a direct relation between high concentrations of metals in water and large cities effluents in a river.

Mainly, the toxicity of HM refers to its ability to form toxic soluble compounds (Amin and Almahasheer 2021). Though some metals are essential for biological processes, chronic exposure could be lethal, inducing kidney and liver malfunction, reproductive and fecundity complications, and nerve injuries in fish (Gaber et al. 2013; Aly and Abouelfadl 2020; Salaah et al. 2021).

According to Table 2, fish accumulate HM relatively to their levels in surrounding, species, and the tendency to store HM is proportional to their concentration in water and exposure period. Consequently, the present higher metal index in *S. galilaeus* could be related to the fish capacity to accumulate or eliminate HM from the tissues. Salaah and El-Gaar (2020) documented the same findings, as S. *galilaeus* recorded higher metals accumulation and MPI in tissues than *O. niloticus*. Moreover, the present accumulation of HM in both fish showed a site dependent response in the present study.

The remarkable fluctuations in bioaccumulation of HM in both fish muscles clearly specify the impact of natural and anthropogenic origins on HM levels in the aquatic system, and the major influence of different pollution sources along the study sampling sites. Consequently, fish inhabited heavily polluted sites of the river will accumulate more HM in their organs and experience more toxic effects than others. So, monitoring levels of HM in fish tissues is crucial to estimate the potential health hazard for humans through the fish intake (Yilmaz et al*.* 2007).

As stated in Table 2, levels of Pb in muscles of both fish have exceeded the recommended guidelines of FAO/WHO (1989) at S3 and FEPA (2003) at S3 and S4 so risk characterization was performed to evaluate the risk of consuming these tissues by a human. The EDI of both fish was within the permissible tolerable daily intake values (USEPA 2014). The THQ values were lower than 1, which assumed no risks from consuming both fish from sampling sites. Noteworthy, the THQ carries out a single HM at a time, which reflects the level of concern not to measure the risk, but food generally includes more than a HM as in the present study, therefore, the hazard index (HI) was considered.

The habitual consumption of both fish from S3 were recorded HI less than 1. So, the present results anticipate possible adverse health risks of both fish habitual consumers from S3. Although, the present data confirmed no carcinogenic risk of consuming any of the two fish from the studied Nile River Islands.

The present study displayed significant histopathological alterations in vital organs of *O. niloticus* and *S. galilaeus*. Melanomacrophage centers (MMCs) were identified in spleen, MMCs is involved in destroying and detoxifying endogenous and exogenous substances such as metals (Matsche et al. 2021). MMCs are used as bioindicators of aquatic pollution and stressors (Manrique et al. 2014). The present increase of MMCs may have resulted from their role in metals detoxification processes, and ability to activate the non-specific immune response against disease and stress (Malins 2018). The relation between MMC responses and the environmental contaminant has been well-cited in wild fish (Steinel and Bolnick 2017).

The environmental stressful conditions induced by HM may give rise to the hepatic lesions, as the liver main function is eliminating toxicants. The present hepatic cellular infiltrations could be an inflammatory response, while the detected hemorrhage along with necrosis may be a consequence of the HM toxicity to the fish liver (Hidayat et al. 2020).

Kaewamatawong et al. (2013) reported similar alterations in the spleen, and liver of fish exposed to HM, degeneration of hepatocytes with vacuolization, and increased number of MMCs were observed within the spleen.

Testis (in spawning stage) were exhibited histopathologic alterations in all sampled fish. Commonly, HM were correlated with reproductive impairments in fish (Salim 2015; Abou Shabana et al. 2008). On the other hand, ovaries of studied fish which collected during spawning season showed many alternations. Similar findings were cited by previous studies concerning impact of HM on fish gonads, reproduction (Bertram et al. 2015; Elgaml et al. 2019), a decline in gonadosomatic index (Gerbron et al. 2014), and reduction of oocyte size (Alquezar et al. 2006). Sangeetha and Aruljothi (2019) observed vacuolation and yolk globles dissolution in the vitellogenic stage. Khillare et al. (2017) detected yolk sac injury and cytoplasm segmentation in mature oocytes, while Shobikhuliatul et al. (2013) distinguished spermatogenesis and lobular structures deterioration with weak sperm production.

The present study emphasizes the ecological risk of various anthropogenic activities on fish collected from four Nile River Islands along the Great Cairo sector. The metal pollution index (MPI) was varied according to sampling site and fish species. Generally, El-Warrak Island recorded the highest metal load in both fish muscles among sites, while El-Zamalek Island recorded the least metal load. Although the target hazard quotient of each heavy metal in the present study indicated no potential health risk for people consuming *O. niloticus* and S. *galilaeus* from all sampling sites, the hazard index recorded possible non-carcinogenic health risk from the habitual consumption of both fish from El-Warrak Island, which is characterized by both agricultural and residential activities. also, the present study documented negligible carcinogenic health risk for consuming *O. niloticus* and *S*. *galilaeus* fish. The present histological lesions observed in different organs of *O. niloticus* and *S*. *galilaeus* anticipates that fish were complying with the direct effects of the heavy metals’ toxicity as well as the secondary effects of stress since the severity of the histopathological lesions followed the MPI of each sampling site.

## Data Availability

The data supporting the findings of this study are available within the article and its supplementary material.
